# Associations of inflammatory biomarkers with brain atrophy and clinical scores in schizophrenia patients with autistic features

**DOI:** 10.1186/s13104-026-07746-1

**Published:** 2026-04-10

**Authors:** Jin Wang, Jie Shen, Yu Pang, Jihui Liu, Lili Zhao, Momo Sun, Yang Yang

**Affiliations:** 1 First Central Hospital of Tianjin Medical University, Tianjin, China; 2 Department of Ultrasound, Tianjin First Central Hospital, Tianjin, China; 3 Department of Nuclear Medicine, Tianjin First Central Hospital, Tianjin, China; 4 Department of General Psychiatry, Tianjin Anding Hospital, Tianjin, China; 5 Department of Ultrasound, Tianjin First Hospital, Tianjin, China; 6 Department of Nuclear Medicine, The Second Affiliated Hospital of Zhengzhou University, Zhengzhou, China

**Keywords:** Schizophrenia, Brain atrophy, Ventricle enlargement, Autism, PAUSS

## Abstract

**Objectives:**

This study explored the schizophrenia patients with autistic traits by analyzing clinical characteristics, inflammatory biomarkers and brain atrophy.

**Results:**

Multiple regression analysis indicated significant associations. Positive symptom scores were linked to higher platelet count, elevated platelet-to-lymphocyte ratio, greater minimum width of the anterior horn of the lateral ventricle, higher Evans index, and male sex. Negative symptom scores correlated positively with older age, higher body mass index, wider third ventricle, higher neutrophil-to-lymphocyte ratio, and male sex. General psychopathology scores rose with older age and greater minimum ventricular width, as did total the Positive and Negative Syndrome Scale (PANSS) scores. The PANSS Autism Severity Score increased with higher neutrophil-to-lymphocyte ratio, greater ventricular widths (minimum anterior horn and third ventricle), older age, larger maximum external cranial diameter, and higher Evans index.

## Introduction

Schizophrenia (SCZ) is a complex disorder marked by psychosis, high comorbidity, and shortened lifespan [1]. Age-related brain changes may contribute to late-onset SCZ [2], while mental illness, brain structure, and metabolism critically shape outcomes [3].

A distinct SCZ subgroup presents with autistic features, exhibiting worse social cognition, functioning, symptom severity, well-being, and treatment response [4, 5]. Although enlarged ventricles are reported in autism [6] and brain-behavior links are established in SCZ [7].

Additionally, immune activation is implicated in SCZ pathogenesis [8], with inflammation and neuroimmune genetics being active research areas [9]. This study therefore aimed to systematically examine the relationships among clinical characteristics, peripheral inflammatory markers, and computed tomography (CT)-based brain atrophy (BA) measures in SCZ patients with autistic traits, and to assess their combined association with clinical severity.

## Materials and methods

### Subjects

This cross-sectional study enrolled 113 SCZ patients (58 males, 55 females; aged 19–82 years) from Tianjin Anding Hospital between September 2023 and January 2024. Exclusion criteria included primary cranial disorders, pregnancy/lactation, non-compliance, and conditions or medications affecting inflammatory markers. Of 126 screened, 13 were excluded (traumatic brain injury = 3, pneumonia = 2, burns = 1, lymphoma = 1, incomplete CT = 6). Following an international consensus [10], participants aged ≥ 60 years were classified as older adults.

### Clinical assessment

Clinical and demographic data (age, sex, illness duration, education, marital status, age of onset, smoking, lifetime suicide attempts, antipsychotic dose in chlorpromazine equivalents, and Body Mass Index (BMI)) were recorded. Symptoms were assessed by two blinded psychiatrists using the 30-item PANSS (score range 30–210, higher scores indicate greater severity) [11, 12], which includes positive, negative, and general psychopathology subscales based on Mohr’s five-factor model. Autistic traits were evaluated with the PANSS Autism Severity Score (PAUSS) [6]. It comprises three subscales measuring difficulties in social interaction (PANSS items N1, N3, N4), communication skills (items N5, N6), and repetitive behaviors (items N7, G5, G15). A cutoff score of 30, as recommended in previous studies [13–15], was used to define clinically relevant autistic features.

###  CT

Two blinded radiologists (> 5 years’ experience) performed all linear measurements. CT scans were acquired on a Philips Ingenuity Core 128-slice scanner with the following parameters: slice thickness/spacing 5 mm, tube voltage/current 120 kV/120 mA, pitch 1.0–1.5 mm, scan time 5 s. Patients were positioned supine, and scanning proceeded upward from the auditory canthus line. Images were reconstructed using CT Viewer (volume, multiplanar, and surface rendering).

The measured parameters included: the maximum width of the anterior horn of the lateral ventricle (MW-AH-LV), the minimum width of the anterior horn of the lateral ventricle (WMIN-AH-LV), third ventricle width (TVW), transverse diameter of the choroid plexus of the lateral ventricle (TD-ChP-LV), transverse diameter of the bilateral caudate nucleus (TD-B-CN), maximum external diameter (MAED) of the body of the LV, maximum external diameter of the cranium (MAED-Cr), maximum internal diameter of the cranium (MAID-Cr), Hackman value (MW-AH-LV + TD-B-CN), ventricular index (TD-ChP-LV/MW-AH-LV), LV body index (MAID-Cr/MAED of the body of the LV), frontal horn index (FHI = MAID-Cr/MW-AH-LV), Evans index (MW-AH-LV/MAID-Cr), and ventricular central index (WMIN-AH-LV/MAID-Cr).

### Laboratory measurements

Morning peripheral venous blood was collected proximate to CT examination. Complete blood count data were used to calculate the neutrophil-to-lymphocyte ratio (NLR), monocyte-to-lymphocyte ratio (MLR), platelet-to-lymphocyte ratio (PLR), and systemic immune-inflammation index (SII).

NLR = neutrophil count (NEU)/lymphocyte count.

MLR = monocyte count/lymphocyte count.

PLR = platelet count/lymphocyte count.

SII = platelet count × neutrophil count/lymphocyte count.

The NLR, MLR, PLR, and SII were used to measure inflammation.

### Statistics

Statistical analysis was conducted via SPSS 26.0. Data normality was assessed with the Kolmogorov‒Smirnov test. Normally distributed data are presented as the means ± standard deviations (SDs), whereas nonnormally distributed data are reported as medians and interquartile ranges (IQRs). Group comparisons were performed using independent samples t-test (normal continuous data), Mann-Whitney U test (non-normal continuous data), or Chi-square test (categorical data), as appropriate. Spearman’s correlation was used to examine the relationships among clinical characteristics, inflammatory biomarkers, BA parameters, and PANSS scores. Multiple linear regression analysis was performed using the backward stepwise method to evaluate the effects of inflammatory biomarkers and BA parameters on the scores. Statistical significance was set at *P* < 0.05.

## Results

### Comparative analysis of clinical characteristics, inflammatory biomarkers and BA parameters across sexes

Table 1 reveals significant sex differences. Males exhibited higher education levels (*P* < 0.001), smoking rates (*P* < 0.01), and suicide attempts (*P* = 0.037), whereas females received greater psychotropic medication (*P* = 0.009). Males also showed elevated NLR (*P* = 0.039), PANSS positive, negative, total scores, and PAUSS (all *P* < 0.05), along with larger brain atrophy parameters including MW-AH-LV, WMIN-AH-LV, TD-ChP-LV, TD-B-CN, MAED-Cr, MAID-Cr, Hackman values (all *P* < 0.01), TVW (*P* = 0.002), and ventricular central indices (*P* = 0.005), indicating more pronounced BA in males.

**Table 1:** Comparative analysis of Clinical Features, inflammatory biomarkers and BA parameters across sexes

Variable	Male (*n* = 58)	Female (*n* = 55)	t/Z/χ^2^	*P*
Age	48.24 ± 13.97	53.04 ± 15.81	1.711	0.090^1^
Onset of the scz	18.00 (10.75-31.00)	26.50 (12.00–38.00)	− 1.270	0.204^2^
Education years	6 (6–9)	6 (0–6)	− 3.864	<0.001^2^
Onset age of SCZ	24.00 ( 20.75–30.25)	26.00 (20.75-31.00)	− 0.242	0.809^2^
History of lifetime suicidal attempts	3.00 (1.00-5.25)	2.00 (1.00–4.00)	− 2.091	0.037^2^
Antipsychotic dosages	344 (200–550)	500 (300–700)	− 2.608	0.009^2^
Marital status				
Unmarried	28 (48.3%)	17 (30.9%)	3.553	0.083^3^
Married	30 (51.7%)	38 (69.1%)		
Smoking status				
Yes	48 (82.8%)	6 (10.9%)	58.408	<0.001^3^
No	10 (17.2%)	49 (89.1%)		
BMI	24.01 (21.91–27.39)	24.31 (21.20–29.00)	− 0.261	0.794 ^2^
PLT	250.86±,63.7	253.93 ± 66.93	0.249	0.804 ^1^
LYM	1.955 (1.73–2.43)	1.900 (1.47–2.27)	− 1.140	0.254 ^2^
NEU	4.42 ± 2.32	3.65 ± 1.94	− 1.905	0.059 ^1^
NLR	3.77 (2.83–3.77)	3.22 (2.49–4.17)	4.253	0.039 ^2^
PLR	122.67 (101.16-148.21)	130.46 (106.04-158.04)	− 1.212	0.225 ^2^
MLR	0.25 (0.19− 148.21)	0.21 (0.16–0.266)	− 1.835	0.066 ^2^
SII	445.58 (352.33-635.41)	427.81 (307.26-602.33)	− 1.086	0.278 ^2^
Positive symptom score	34 (31-37.25)	31 (29–32)	− 4.172	<0.001 ^2^
Negative symptom score	31 (28-36.25)	30 (27–32)	− 2.159	0.031 ^2^
General psychopathological symptoms score	68 (65.75–72.25)	68 (66–69)	− 1.517	0.159 ^2^
Total score	134.91 ± 15.47	127.6 ± 5.56	− 3.308	0.001 ^1^
PAUSS	37.45 ± 6.27	35.24 ± 3.64	− 2.276	0.025 ^1^
MW-AH-LV (mm)	3.52 ± 0.38	3.33 ± 0.4	− 2.609	0.01 ^1^
TD-B-CN (mm)	1.72 (1.53–1.95)	1.34 (1.22–1.72)	− 4.657	<0.01 ^2^
WMIN-AH-LV (mm)	1.79 ± 0.31	1.54 ± 0.29	− 4.458	<0.01 ^1^
TVW (mm)	0.69 (0.58–0.80)	0.57 (0.47–0.74)	− 3.037	0.002 ^2^
TD-ChP-LV (mm)	6.02 ± 0.43	5.52 ± 0.58	− 5.206	<0.01 ^1^
MAED of body of LV (mm)	2.65 ± 0.37	2.59 ± 0.37	− 0.763	0.447 ^1^
MAED-Cr (mm)	14.81 ± 0.59	14.27 ± 0.5	− 5.299	<0.01 ^1^
MAID-Cr (mm)	13.29 ± 0.6	12.64 ± 0.54	− 6.03	<0.01 ^1^
Hackman value	5.29 ± 0.53	4.81 ± 0.7	− 4.11	<0.01 ^1^
Ventricular index	1.72 ± 0.17	1.68 ± 0.24	− 1.174	0.243 ^1^
The ventricular central indexs	0.14 ± 0.02	0.12 ± 0.02	− 2.834	0.005 ^1^
LV body index	5.11 ± 0.69	4.97 ± 0.72	− 1.044	0.299 ^1^
FHI	3.74 (3.58–3.99)	3.75 (3.58–4.13)	− 0.345	0.730 ^2^
Evans index	0.26 ± 0.03	0.26 ± 0.03	− 0.251	0.803 ^1^

^1^Independent samples t test

^2^Mann‒Whitney U test

^3^Chi-square test

Data are presented as median (interquartile range, IQR) for non-normally distributed continuous variables, mean ± standard deviation, SD for normally distributed continuous variables, and number (%) for categorical variables. *IQR* Interquartile range; *SD* Standard deviation. *BA* Brain atrophy; *SCZ* Schizophrenia; *BMI* Body mass index; *PLT* Platelet count; *LYM* Lymphocyte count; *NEU* Neutrophil count; *NLR* NEU/LYM; *PLR* PLT/LYM; *MLR* Monocyte count/LYM; *SII* PLT ×NEU/LYM; *PANSS* Positive and Negative Syndrome Scale; *PAUSS* PANSS Autism Severity Score; *MW-AH-LV* Maximum width of the anterior horn of the lateral ventricle; *TD-B-CN* Transverse diameter of the bilateral caudate nucleus; *WMIN-AH-LV* Minimum width of the anterior horn of the lateral ventricle; *TVW* Third ventricle width; *TD-ChP-LV* Transverse diameter of the choroid plexus of the lateral ventricle; *MAED* Maximum external diameter; *MAED-Cr* Maximum external diameter of cranium; *Hackman value* MW-AH-LV + TD-B-CN, the ventricular index = TD-ChP-LV/MW-AH-LV, the LV body index=MAID-Cr/MAED of the body of the LV, the frontal horn index, FHI=MAID-Cr/MW-AH-LV, the Evans index = MW-AH-LV/MAID-Cr, the ventricular central indexs=WMIN-AH-LV/MAID-Cr

### Comparative analysis of inflammatory biomarkers and BA parameters across age groups

PLT was lower in patients over 60 (*P* = 0.028). The older group showed higher negative symptom, total PANSS, and PAUSS scores (*P* < 0.01), along with greater BA parameters: WMIN-AH-LV, TVW, TD-ChP-LV, MAED of LV body, Evans index, ventricular central index (all *P* < 0.01), MW-AH-LV (*P* = 0.004), MAID-Cr (*P* = 0.001), TD-B-CN (*P* = 0.001), Hackman value (*P* = 0.001), ventricular index (*P* = 0.028), and FHI (*P* = 0.022).

### Correlation analysis of disease duration, clinical characteristics, inflammatory biomarkers, and BA parameters

Longer disease duration correlated positively with total score, general psychopathology, PAUSS, MW-AH-LV, TD-B-CN, TVW, TD-ChP-LV, MAED-Cr, ventricular index, LV body index, age, and BMI (all *P* < 0.01). PAUSS severity increased with disease progression (*r* = 0.360, *P* < 0.01). Negative correlations were observed with PLT, NEU, Hackman value, FHI, Evans index, education years, and onset age of SCZ (all *P* < 0.05). Longer disease duration was associated with lower inflammatory markers but greater ventricular dilation (*P* < 0.01) (Table 2).

**Table 2:** Correlation analysis of disease duration, Clinical Features, inflammatory biomarkers, and BA parameters

	Disease duration
Age	*r* = 0.812, *P* < 0.01
Education years	*r* = − 0.246, *P* < 0.01
Onset of SCZ	*r* = − 0.320, *P* < 0.01
History of lifetime suicidal attempts	*r* = 0.027, *P* = 0.774
Antipsychotic dosages	*r* = 0.008, *P* = 0.931
Marital status	*r* = 0.065, *P* = 0.497
Smoking status	*r* = 0.006, *P* = 0.948
BMI	*r* = 0.200, *P* = 0.033
PLT	*r* = − 0.333, *P* < 0.01
LYM	*r* = − 0.148, *P* = 0.118
NEU	*r* = − 0.197, *P* = 0.036
NLR	*r* = − 0.126, *P* = 0.184
PLR	*r* = − 0.134, *P* = 0.159
MLR	*r* = 0.145, *P* = 0.124
SII	*r* = − 0.172, *P* = 0.068
Positive symptom score	*r* = − 0.083, *P* = 0.601
Negative symptom score	*r* = 0.137, *P* = 0.148
General psychopathological symptoms score	*r* = 0.526, *P* < 0.01
Total score	*r* = 0.232, *P* = 0.013
PAUSS	*r* = 0.360, *P* < 0.01
MW-AH-LV (mm)	*r* = 0.467, *P* < 0.01
TD-B-CN (mm)	*r* = 0.409, *P* < 0.01
WMIN-AH-LV (mm)	*r* = − 0.354, *P* < 0.01
TVW (mm)	*r* = 0.799, *P* < 0.01
TD-ChP-LV (mm)	*r* = 0.406, *P* < 0.01
MAED of body of LV (mm)	*r* = 0.115, *P* = 0.227
MAED-Cr (mm)	*r* = 0.360, *P* < 0.01

*BA* Brain atrophy; *SCZ* Schizophrenia; *BMI* Body mass index; *PLT* Platelet count; *LYM* Lymphocyte count; *NEU* neutrophil count; *NLR* NEU/LYM; *PLR* PLT/LYM; *MLR* Monocyte count/LYM; *SII* PLT ×NEU/LYM; *PANSS* Positive and Negative Syndrome Scale; *PAUSS* PANSS Autism Severity Score; *MW-AH-LV* Maximum width of the anterior horn of the lateral ventricle; *TD-B-CN* Transverse diameter of the bilateral caudate nucleus; *WMIN-AH-LV* Minimum width of the anterior horn of the lateral ventricle; *TVW* Third ventricle width; *TD-ChP-LV* transverse diameter of the choroid plexus of the lateral ventricle; *MAED* Maximum external diameter; *MAED-Cr* Maximum external diameter of cranium; *Hackman value* MW-AH-LV + TD-B-CN, the ventricular index = TD-ChP-LV/MW-AH-LV, the LV body index=MAID-Cr/MAED of the body of the LV, the frontal horn index, FHI=MAID-Cr/MW-AH-LV, the Evans index = MW-AH-LV/MAID-Cr, the ventricular central indexs=WMIN-AH-LV/MAID-Cr

### Correlation analysis of clinical characteristics, inflammatory biomarkers, and BA parameters with PANSS/PAUSS scores

Significant correlations were identified. Positive symptoms correlated with smoking and suicide attempts. Negative symptoms correlated positively with age, illness duration, and smoking, but negatively with education. The PANSS total and general psychopathology scores were positively associated with age, illness duration, and smoking. PAUSS was positively correlated with age, illness duration, and smoking.

Inflammatory biomarkers showed that negative symptoms were positively associated with MLR but negatively with PLT and LYM. PAUSS was negatively correlated with PLT.

Regarding BA parameters, positive symptoms correlated positively with WMIN-AH-LV, TD-ChP-LV, ventricular index, and ventricular central index. Negative symptoms correlated positively with TD-B-CN, WMIN-AH-LV, TVW, ventricular central index, TD-ChP-LV, and Hackman value. General psychopathology correlated positively with ventricular index, FHI, and Evans index. The total score correlated positively with TD-B-CN, WMIN-AH-LV, TVW, TD-ChP-LV, ventricular index, and ventricular central indices. PAUSS correlated positively with TD-B-CN, WMIN-AH-LV, TVW, TD-ChP-LV, Hackman value, and ventricular central indices. Table 3.

**Table 3:** Correlation analysis of Clinical Features, inflammatory biomarkers, and BA parameters and PANSS or PAUSS

	Positive symptom score	Negative symptom score	General psychopathological symptoms score	Total score	PAUSS
Age	*r* = 0.121, *P* = 0.201	*r* = 0.457, *P <* 0.001	*r* = 0.362, *P <* 0.001	*r* = 0.310, *P* = 0.001	*r* = 0.188, *P* = 0.046
Disease duration	*r* = 0.145, *P* = 0.126	*r* = 0.530, *P <* 0.001	*r* = 0.463, *P <* 0.001	*r* = 0.379, *P <* 0.001	*r* = 0.265, *P* = 0.005
Education years	*r* = − 0.001, *P* = 0.991	*r* = − 0.265, *P* = 0.005	*r* = − 0.262, *P* = 0.005	*r* = − 0.139, *P* = 0.141	*r* = − 0.100, *P* = 0.292
Onset age of SCZ	*r* = − 0.067, *P* = 0.481	*r* = − 0.135, *P* = 0.155	*r* = − 0.175, *P* = 0.064	*r* = − 0.126, *P* = 0.183	*r* = − 0.117, *P* = 0.215
History of lifetime suicidal attempts	*r* = 0.288, *P* = 0.002	*r* = 0.043, *P* = 0.650	*r* = 0.120, *P* = 0.204	*r* = 0.122, *P* = 0.196	*r* = − 0.002, *P* = 0.982
Antipsychotic dosages	*r* = 0.134, *P* = 0.157	*r* = − 0.105, *P* = 0.273	*r* = − 0.055, *P* = 0.565	*r* = − 0.108, *P* = 0.255	*r* = − 0.029, *P* = 0.759
Marital status	*r* = 0.029, *P* = 0.763	*r* = − 0.006, *P* = 0.949	*r* = − 0.018, *P* = 0.852	*r* = 0.066, *P* = 0.485	*r* = 0.044, *P* = 0.646
Smoking status	*r* = 0.379, *P <* 0.001	*r* = 0.319, *P* = 0.001	*r* = 0.268, *P* = 0.004	*r* = 0.301, *P* = 0.001	*r* = 0.106, *P* = 0.264
BMI	*r* = 0.025, *P* = 0.791	*r* = 0.220, *P* = 0.019	*r* = 0.157, *P* = 0.096	*r* = 0.151, *P* = 0.110	*r* = 0.128, *P* = 0.175
PLT	*r* = − 0.113, *P* = 0.231	*r* = − 0.255, *P* = 0.006	*r*=− 0.031, *P* = 0.747	*r* = − 0.142, *P* = 0.134	*r* = − 0.190, *P* = 0.043
LYM	*r* = − 0.032, *P* = 0.737	*r* = − 0.186, *P* = 0.049	*r*=− 0.057, *P* = 0.549	*r* = − 0.086, *P* = 0.367	*r* = − 0.159, *P* = 0.093
NEU	*r* = − 0.069, *P* = 0.47	*r* = − 0.112, *P* = 0.236	*r* = 0.012, *P* = 0.899	*r* = − 0.059, *P* = 0.532	*r* = − 0.114, *P* = 0.228
NLR	*r* = 0.008, *P* = 0.932	*r* = − 0.051, *P* = 0.595	*r* = 0.032, *P* = 0.734	*r* = − 0.01, *P* = 0.917	*r* = − 0.051, *P* = 0.594
PLR	*r* = 0.016, *P* = 0.866	*r* = − 0.064, *P* = 0.498	*r* = 0.026, *P* = 0.782	*r* = − 0.018, *P* = 0.853	*r* = − 0.036, *P* = 0.707
MLR	*r* = 0.064, *P* = 0.501	*r* = 0.210, *P* = 0.026	*r* = 0.098, *P* = 0.303	*r* = 0.127, *P* = 0.18	*r* = 0.125, *P* = 0.188
SII	*r* = − 0.012, *P* = 0.9	*r* = − 0.101, *P* = 0.287	*r* = 0.027, *P* = 0.776	*r* = − 0.035, *P* = 0.716	*r* = − 0.09, *P* = 0.345
MW-AH-LV (mm)	*r* = 0.005, *P* = 0.958	*r* = 0.091, *P* = 0.339	*r*=− 0.169, *P* = 0.073	*r* = − 0.034, *P* = 0.724	*r* = 0.056, *P* = 0.553
TD-B-CN (mm)	*r* = 0.157, *P* = 0.097	*r* = 0.429, *P <* 0.001	*r* = 0.092, *P* = 0.334	*r* = 0.274, *P* = 0.003	*r* = 0.316, *P* = 0.001
WMIN-AH-LV (mm)	*r* = 0.333, *P <* 0.001	*r* = 0.366, *P <* 0.001	*r* = 0.137, *P* = 0.149	*r* = 0.330, *P <* 0.001	*r* = 0.288, *P* = 0.002
TVW (mm)	*r* = 0.184, *P* = 0.050	*r* = 0.413, *P <* 0.001	*r* = 0.138, *P* = 0.144	*r* = 0.331, *P <* 0.001	*r* = 0.348, *P <* 0.001
TD-ChP-LV (mm)	*r* = 0.250, *P* = 0.008	*r* = 0.208, *P* = 0.027	*r* = 0.152, *P* = 0.109	*r* = 0.247, *P* = 0.008	*r* = 0.237, *P* = 0.012
MAED of body of LV (mm)	*r* = 0.068, *P* = 0.471	*r* = 0.175, *P* = 0.063	*r* = 0.02, *P* = 0.834	*r* = 0.117, *P* = 0.218	*r* = 0.11, *P* = 0.248
MAED-Cr (mm)	*r* = 0.158, *P* = 0.095	*r* = 0.08, *P* = 0.401	*r* = 0.027, *P* = 0.778	*r* = 0.109, *P* = 0.249	*r* = 0.174, *P* = 0.066
MAID-Cr (mm)	*r* = 0.121, *P* = 0.2	*r* = 0.02, *P* = 0.83	*r* = 0.034, *P* = 0.72	*r* = 0.085, *P* = 0.373	*r* = 0.069, *P* = 0.47
Hackman value	*r* = 0.095, *P* = 0.315	*r* = 0.308, *P* = 0.001	*r*=− 0.049, *P* = 0.608	*r* = 0.141, *P* = 0.136	*r* = 0.220, *P* = 0.019
Ventricular index	*r* = 0.187, *P* = 0.047	*r* = 0.071, *P* = 0.452	*r* = 0.305, *P* = 0.001	*r* = 0.234, *P* = 0.013	*r* = 0.128, *P* = 0.178
The ventricular central indexs	*r* = 0 0.296, *P* = 0.001	*r* = 0.365, *P <* 0.001	*r* = 0.12, *P* = 0.204	*r* = 0.304, *P* = 0.001	*r* = 0.269, *P* = 0.004
LV body index	*r* = − 0.027, *P* = 0.773	*r* = − 0.168, *P* = 0.075	*r* = 0.004, *P* = 0.969	*r* = − 0.082, *P* = 0.387	*r* = − 0.088, *P* = 0.356
FHI	*r* = 0.049, *P* = 0.607	*r* = − 0.095, *P* = 0.315	*r* = 0.219, *P* = 0.02	*r* = 0.082, *P* = 0.389	*r* = − 0.042, *P* = 0.659
Evans index	*r* = − 0.051, *P* = 0.592	*r* = 0.096, *P* = 0.313	*r*=− 0.199, *P* = 0.035	*r* = − 0.072, *P* = 0.446	*r* = 0.032, *P* = 0.736

*BA* Brain atrophy; *BMI* Body mass index; *PLT* Platelet count; *LYM* Lymphocyte count; *NEU* Neutrophil count; *NLR* NEU/LYM; *PLR* PLT/LYM; *MLR* Monocyte count/LYM; *SII* PLT ×NEU/LYM; *PANSS* Positive and Negative Syndrome Scale; *PAUSS* PANSS Autism Severity Score; *MW-AH-LV* Maximum width of the anterior horn of the lateral ventricle; *TD-B-CN* transverse diameter of the bilateral caudate nucleus; *WMIN-AH-LV* minimum width of the anterior horn of the lateral ventricle; *TVW* Third ventricle width; *TD-ChP-LV* Transverse diameter of the choroid plexus of the lateral ventricle; *MAED* Maximum external diameter; *MAED-Cr* Maximum external diameter of cranium; *Hackman value* MW-AH-LV + TD-B-CN, the ventricular index = TD-ChP-LV/MW-AH-LV, the LV body index=MAID-Cr/MAED of the body of the LV, the frontal horn index, FHI=MAID-Cr/MW-AH-LV, the Evans index = MW-AH-LV/MAID-Cr, the ventricular central indexs=WMIN-AH-LV/MAID-Cr

### The combination of clinical characteristics, BA parameters and inflammatory biomarkers accurately predicted the PANSS and PAUSS scores

**Fig. 1:**
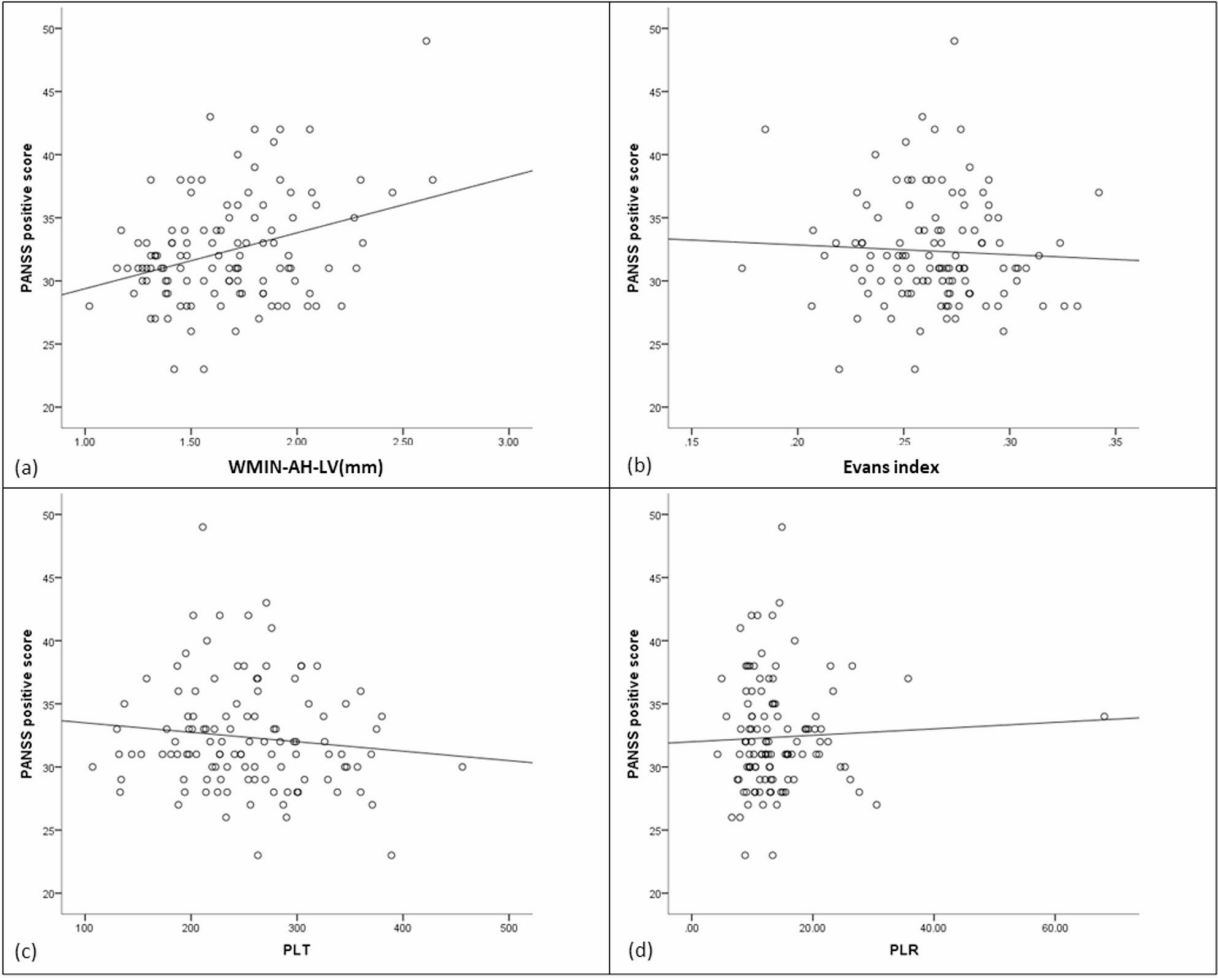
Scatterplot for the association between WMIN-AH-LV (mm) (**a**), Evans index (**b**), PLT (**c**), PLR (**d**) and positive score. *WMIN-AH-LV* Minimum width of the anterior horn of the lateral ventricle; *PLT* Platelet count; *LYM* Lymphocyte count; *PLR* PLT/LYM; *MW-AH-LV* Maximum width of the anterior horn of the lateral ventricle; *MAID-Cr* Maximum internal diameter of cranium; the Evans index = MW-AH-LV/MAID-Cr

A significant multiple regression model for positive symptoms was identified (F = 6.946, *P* < 0.001, R^2^=0.327). Using the backward method with clinical characteristics, BA parameters, and inflammatory biomarkers as independent variables (Table 4), positive scores showed a negative association with PLT (*P* = 0.036) and Evans index (*P* = 0.001), and a positive association with WMIN-AH-LV (*P* < 0.001), PLR (*P* = 0.009), and male sex (*P* = 0.012) (Fig. 1).

**Table 4:** The multiple regression analysis of Positive symptom score

Variable	β	SE	t	*P*
Constant	32.428	4.281		
WMIN-AH-LV (mm)	8.270	2.317	3.570	0.001
Evans index	− 57.126	15.484	− 3.689	< 0.001
PLT	− 0.025	0.007	− 2.621	0.020
PLR	0.027	0.009	2.346	0.027
Sex	2.929	0.836	3.504	0.001

*WMIN-AH-LV* Minimum width of the anterior horn of the lateral ventricle; *PLT* Platelet count; *LYM* Lymphocyte count; *PLR* PLT/LYM; *MW-AH-LV* Maximum width of the anterior horn of the lateral ventricle; *MAID-Cr* maximum internal diameter of cranium; the Evans index = MW-AH-LV/MAID-Cr

**Fig. 2:**
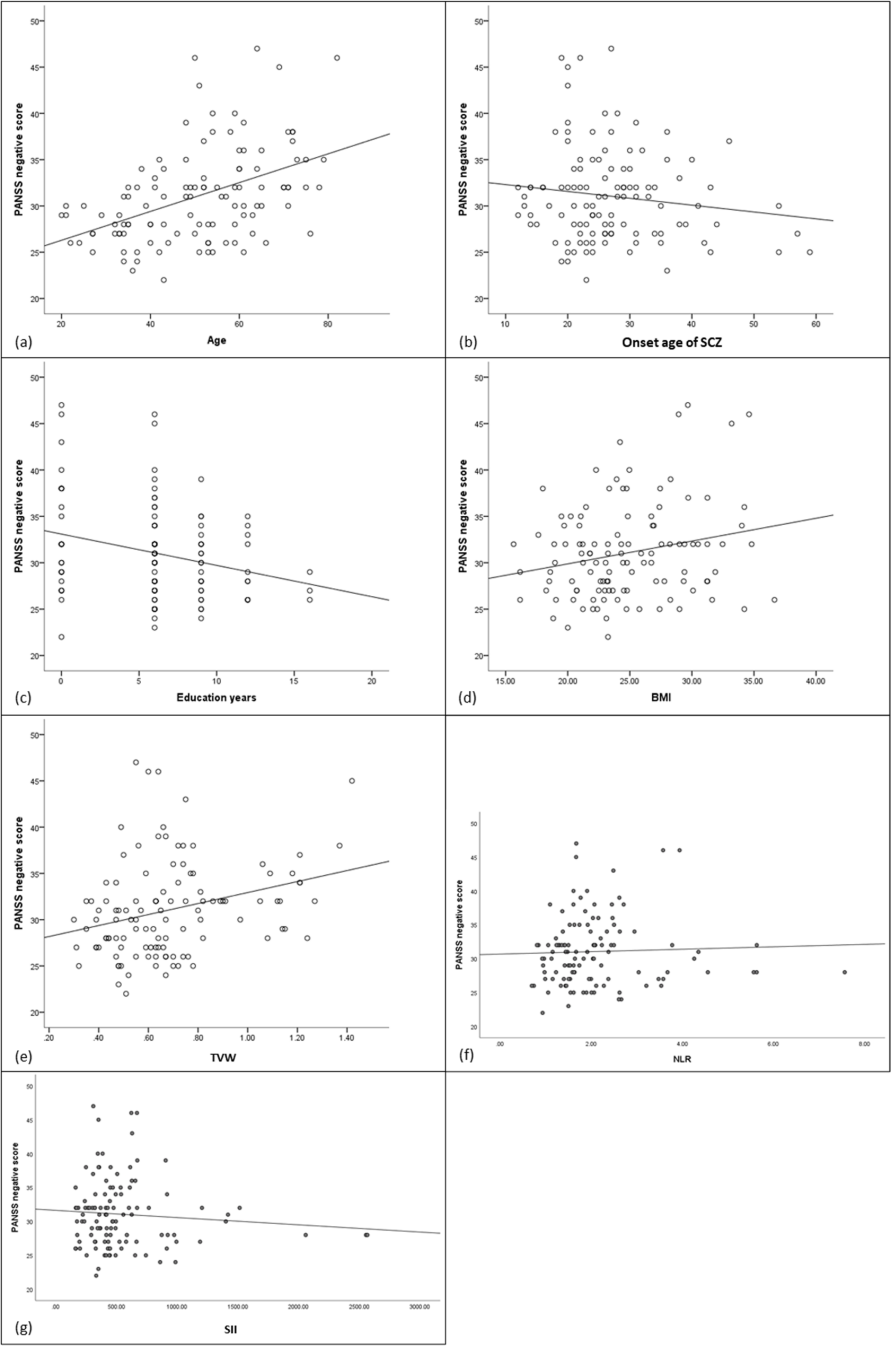
Scatterplot for the association between Age (**a**), Onset age of SCZ (**b**), Education years (**c**), BMI (**d**), TVW (**e**), NLR (**f**), SII (**g**) and PANSS negative score. *SCZ* Schizophrenia; *BMI* Body Mass Index; *TVW* Third ventricle width; *LYM* Lymphocyte count; *NEU* Neutrophil count; *NLR* NEU/LYM; *PLT* Platelet count; *SII* PLT ×NEU/LYM

The regression model for negative symptoms was significant (R^2^=0.518, F = 10.097, *P* < 0.001) (Table 5). Negative symptoms decreased with higher SII, more education years, and later onset age of SCZ, but increased with higher NLR, wider TVW, older age, male sex, and higher BMI (Fig. 2).

**Table 5:** The multiple regression analysis of Negative symptom score

Variable	β	SE	t	*P*	VIF
Constant	20.538	1.665			
Age	0.178	0.035	5.098	< 0.001	2.380
Onset age of SCZ	− 0.122	0.042	− 2.938	0.004	1.313
Education years	− 0.387	0.095	− 4.060	< 0.001	1.283
BMI	0.203	0.082	2.482	0.015	1.219
TVW	4.260	2.106	2.379	0.023	2.157
NLR	1.317	0.564	2.336	0.022	1.276
SII	− 0.006	0.002	− 2.205	0.019	1.670
Sex	3.752	0.974	3.853	< 0.001	2.067

*SCZ* Schizophrenia; *BMI* Body Mass Index; *TVW* Third ventricle width; *LYM* Lymphocyte count; *NEU* Neutrophil count; *NLR* NEU/LYM; *PLT* Platelet count; *SII* PLT ×NEU/LYM

**Fig. 3:**
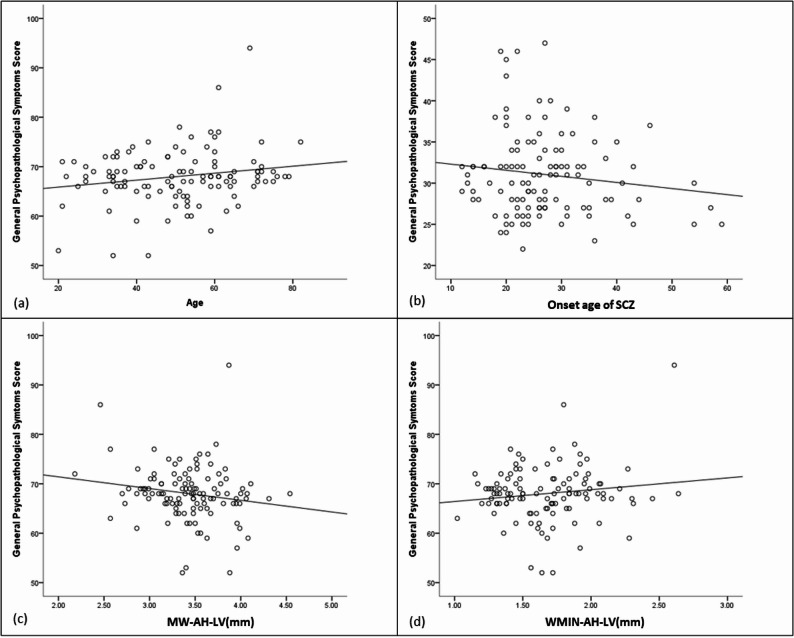
Scatterplot for the association between Age (**a**), Onset age of SCZ (**b**), MW-AH-LV (mm) (**c**), WMIN-AH-LV (mm) (**d**), and General psychopathological symptoms score. *SCZ* Schizophrenia; *MW-AH-LV* Maximum width of the anterior horn of the lateral ventricle; *WMIN-AH-LV* Minimum width of the anterior horn of the lateral ventricle; the ventricular central indexs=WMIN-AH-LV/MAID-Cr; *MAED-Cr* maximum external diameter of cranium

The regression model for general psychopathology was significant (R^2^=0.195, F = 6.312, *P* < 0.001) (Table 6). The score decreased with larger MW-AH-LV, higher ventricular central indices, and later onset age of SCZ, but increased with larger WMIN-AH-LV and older age (Fig. 3).

**Table 6:** The multiple regression analysis of General psychopathological symptoms score

Variable	β	SE	t	*P*	VIF
Constant	81.259	4.695			
Age	0.130	0.043	3.042	0.003	1.761
Onset age of SCZ	− 0.125	0.055	− 2.272	0.025	1.131
MW-AH-LV (mm)	− 6.894	1.604	− 4.299	< 0.001	1.804
WMIN-AH-LV (mm)	24.922	6.906	3.609	< 0.001	3.054
The ventricular central indexs	− 267.867	89.180	− 3.004	0.003	3.527

*SCZ* Schizophrenia; *MW-AH-LV* Maximum width of the anterior horn of the lateral ventricle; *WMIN-AH-LV* minimum width of the anterior horn of the lateral ventricle; the ventricular central indexs=WMIN-AH-LV/MAID-Cr; *MAED-Cr* Maximum external diameter of cranium

**Fig. 4:**
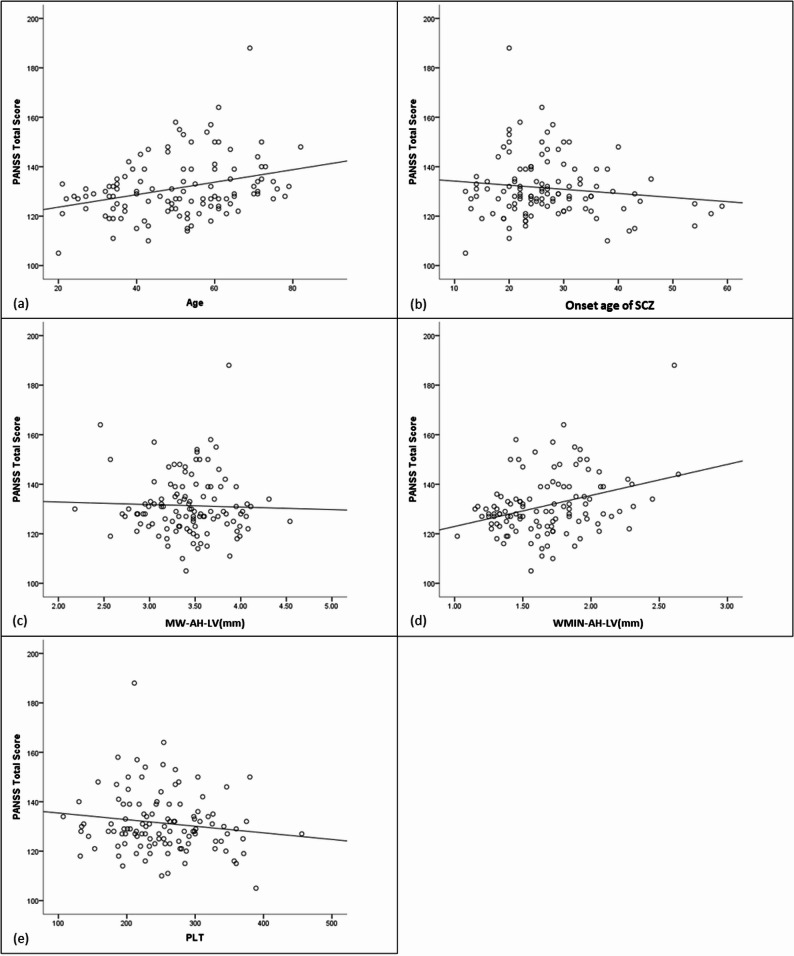
Scatterplot for the association between Age (**a**), Onset age of SCZ (**b**), MW-AH-LV (**c**), WMIN-AH-LV (**d**) PLT (**e**) and Total score. *SCZ* Schizophrenia; *MW-AH-LV* Maximum width of the anterior horn of the lateral ventricle; *WMIN-AH-LV* Minimum width of the anterior horn of the lateral ventricle; the ventricular central indexs=WMIN-AH-LV/MAID-Cr; *MAED-Cr* Maximum external diameter of cranium; *PLT* Platelet count

The regression model for total score was significant (R^2^=0.364, F = 8.865, *P* < 0.001) (Table 7). The score decreased with higher PLT, larger MW-AH-LV, higher ventricular central indices, and later onset age of SCZ, but increased with larger WMIN-AH-LV and older age (Fig. 4).

**Table 7:** The multiple regression analysis of Total score

Variable	β	SE	t	*P*	VIF
Constant	148.079	9.651			
Age	0.386	0.085	4.518	< 0.001	1.875
Onset age of SCZ	− 0.296	0.108	− 2.748	0.007	1.157
MW-AH-LV (mm)	− 13.704	3.218	− 4.258	< 0.001	1.976
WMIN-AH-LV (mm)	51.837	15.662	3.310	0.001	5.276
The ventricular central indexs	− 534.701	189.912	− 2.816	0.006	5.033
PLT	− 0.024	0.011	− 2.049	0.043	1.611
Sex	6.752	2.403	2.809	0.006	1.652

*SCZ* schizophrenia; *MW-AH-LV* Maximum width of the anterior horn of the lateral ventricle; *WMIN-AH-LV* minimum width of the anterior horn of the lateral ventricle; the ventricular central indexs=WMIN-AH-LV/MAID-Cr; *MAED-Cr* Maximum external diameter of cranium; *PLT* platelet count

**Fig. 5:**
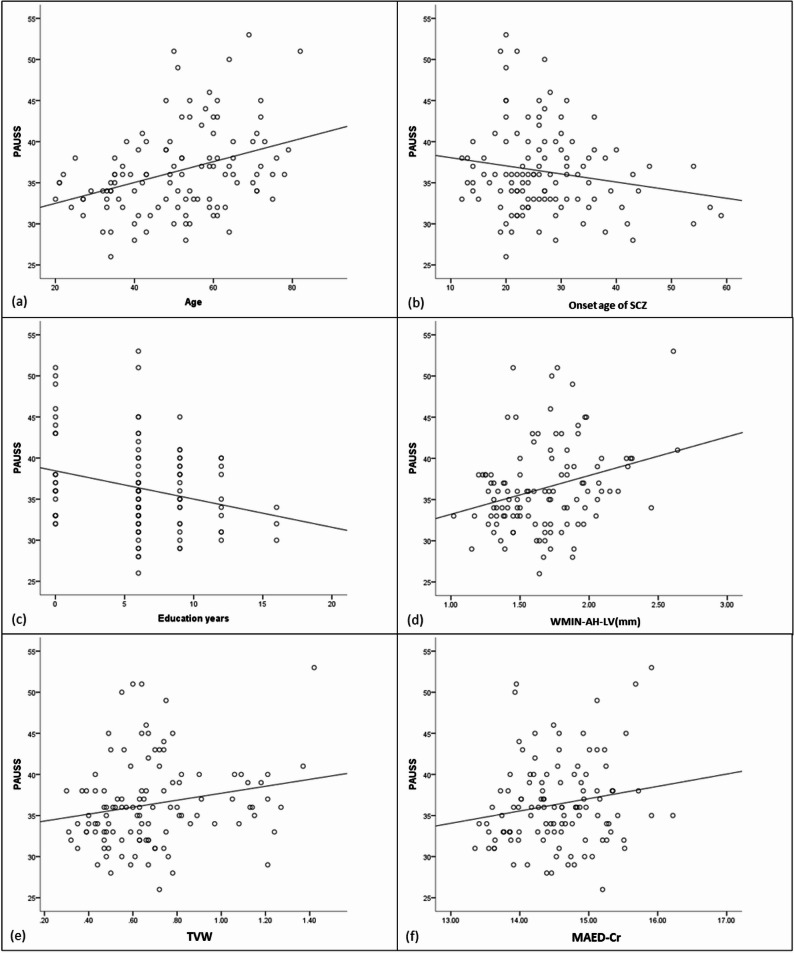
Scatterplot for the association between Age (**a**), Onset age of SCZ (**b**), Education years (**c**), WMIN-AH-LV (mm) (**d**), TVW (**e**), MAED-Cr (**f**), Evans index (**g**), SII (**h**), NLR (**i**) and PAUSS. *SCZ* Schizophrenia; *PLT* Platelet count; *MW-AH-LV* Maximum width of the anterior horn of the lateral ventricle; *WMIN-AH-LV* Minimum width of the anterior horn of the lateral ventricle; *TVW* Third ventricle width; the ventricular central indexs=WMIN-AH-LV/MAID-Cr; *MAED-Cr* Maximum external diameter of cranium; *NEU* neutrophil count; *NLR* NEU/LYM; *PLT* Platelet count; *SII* PLT ×NEU/LYM

The regression model for PAUSS was significant (R^2^=0.391, F = 11.086, *P* < 0.001) (Table 8). PAUSS decreased with higher SII, higher ventricular central indices, later onset age of SCZ, and more education years. PAUSS increased with higher NLR, larger MAED-Cr, larger WMIN-AH-LV, wider TVW, higher Evans index, older age, and male sex (Fig. 5).

**Table 8:** The multiple regression analysis of PAUSS

Variable	β	SE	t	*P*	VIF
Constant	− 32.035	21.227			
Age	0.166	0.029	5.750	< 0.001	1.227
Onset age of SCZ	− 0.127	0.045	− 2.805	0.006	1.167
Education years	− 0.386	0.107	− 3.618	< 0.001	1.200
MW-AH-LV (mm)	− 50.027	18.897	− 2.211	0.017	2.874
WMIN-AH-LV (mm)	74.528	30.175	1.971	0.032	6.524
TVW	5.315	1.809	2.126	0.022	5.883
The ventricular central indexs	− 825.493	379.761	− 1.846	0.045	1.625
MAED-Cr	1.683	0.742	2.267	0.025	1.334
Evans index	515.407	211.560	2.109	0.021	5.503
SII	− 0.006	0.002	− 1.984	0.035	3.886
NLR	1.189	0.595	2.007	0.038	1.090
Sex	3.103	0.925	3.356	0.001	1.392

*SCZ* Schizophrenia; *PLT* Platelet count; *MW-AH-LV* Maximum width of the anterior horn of the lateral ventricle; *WMIN-AH-LV* Minimum width of the anterior horn of the lateral ventricle; *TVW* Third ventricle width; the ventricular central indexs=WMIN-AH-LV/MAID-Cr; *MAED-Cr* Maximum external diameter of cranium; the Evans index = MW-AH-LV/MAID-Cr; *LYM* Lymphocyte count; *NEU* Neutrophil count; *NLR* NEU/LYM; *PLT*, Platelet count; *SII* PLT ×NEU/LYM

## Discussion

Consistent with our findings, prior research has linked elevated BMI to negative symptoms and depression [16]. Epidemiological data show higher smoking rates in male SCZ patients [17], with smokers exhibiting more severe symptoms [18], potentially via reduced gray matter and elevated cytokines [19]. In our study, smoking correlated positively with PANSS scores but did not enter the final regression models.

Earlier onset is associated with more severe symptoms [20, 21], and may reflect prefrontal gray matter deficits [22, 23]. Our findings align, showing significant correlations between onset age and both PANSS and PAUSS scores.

Depressive comorbidity in SCZ (30–70%) varies by sex [24, 25].Our results are consistent with prior work linking learning potential to cognitive and social functioning [26].

Compared with magnetic resonance imaging (MRI), brain measurements from CT scans are significantly correlated with clinical outcomes in patients with SCZ, despite the lower spatial resolution of CT. A previous study [27] revealed that CT has superior data accessibility, especially for large-scale clinical studies. It also offers shorter scan times and greater patient convenience [28]. For patients without focal neurological symptoms, routine structural neuroimaging may not be necessary. When imaging is needed, CT has comparable diagnostic efficacy to MRI and is suitable as a first-line modality [29].

Ventricular enlargement in SCZ is well-established [7, 30], also observed in high-risk populations [31] and ASD [5, 32]. This study revealed significant correlations between brain atrophy parameters and clinical symptom scores, particularly involving the lateral ventricle, third ventricle, and caudate nucleus. Both WMIN-AH-LV and TVW strongly influenced all scores. These findings are consistent with previous reports and confirm a robust link between schizophrenia with autistic features (PAUSS) and enlargement of the lateral ventricle, third ventricle, and caudate nucleus.

Longer illness duration and age > 60 were associated with greater BA [33], possibly influenced by antipsychotics [34].Ventriculomegaly correlated with negative symptoms and cognitive impairment [35], supported by findings in FEP [36, 37].

The correlations between the negative symptom score, PAUSS score, and BA parameters remained consistent. The PAUSS is positively linked to the caudate nucleus, LV, ChP, and third ventricle. The NLR also showed significant correlations in the regression analyses.

Hypothalamic atrophy may underlie third ventricle enlargement in ASD [38], a hypothesis consistent with our findings. ChP enlargement correlated with multiple symptom domains, aligning with reports of ChP dysfunction, chronic inflammation, and hormonal influences on sex differences in BA [39–42].Caudate diameter correlated positively with negative symptoms and PAUSS, supporting its inclusion in regression models.

Inflammation–SCZ links are well-documented [43, 44] has consistently shown a link between various bacterial and viral infections and the occurrence of mental disorders. Elevated levels of diverse autoimmune antibodies have been detected in individuals diagnosed with SCZ [44]. Our finding that negative symptoms correlated positively with MLR and negatively with PLT/LYM, and that NLR predicted both negative symptoms and PAUSS, aligns with prior reports of elevated NLR, PLR, and MLR in SCZ [45].

SII, though inversely associated with symptom severity in our models, has been linked to PANSS scores in previous work [46]. After confounder adjustment, NLR remained consistently associated with clinical outcomes, whereas SII uniquely predicted global and general psychopathology scores [47].

## Limitations

First, the cross-sectional design precludes causal inference. Second, the single-center sample with modest size may limit generalizability. Third, CT linear measurements, though practical, lack MRI’s spatial resolution for detailed volumetric analysis. Fourth, multiple testing corrections were not applied to preserve statistical power for planned, independent hypotheses across distinct variable domains, which increases Type I error risk. Fifth, peripheral inflammatory indices (e.g., NLR, PLR, SII) are indirect proxies susceptible to unmeasured clinical or temporal influences. Finally, potential confounders, including detailed antipsychotic medication history, were not fully accounted for.In summary, limitations include study design, sample, methods, and unmeasured confounders. Future longitudinal, multi-center studies with advanced neuroimaging and specific immune markers are needed.

## Conclusion

In SCZ patients with autistic features, clinical characteristics, inflammatory biomarkers, and BA show positive correlations with PANSS or PAUSS scores. These readily available clinical and imaging parameters may serve as adjunctive, objective indicators that could aid in the clinical profiling of this distinct subgroup, warranting further investigation into their utility.

## Data Availability

The clinical data supporting the findings of this study are not publicly available due to patient privacy concerns but can be made available by the corresponding author after signing a data use agreement and with the permission of the institutional ethics committee.

## Abbreviations

SCZ: Schizophrenia
BA: Brain atrophy
CT: Computed tomography
PLT: Platelet count
NEU: Neutrophil count
NLR: Lymphocyte ratio
MLR: Monocyte-to-lymphocyte ratio
PLR: Platelet-to-lymphocyte ratio
SII: Systemic inflammation index
PANSS: Positive and Negative Syndrome Scale
PAUSS: PANSS Autism Severity Score
BMI: Body Mass Index
LYM: Lymphocyte
WMIN-AH-LV: Minimum width of the anterior horn of the lateral ventricle
TD-B-CN: Transverse diameter of the bilateral caudate nucleus
TVW: Third Ventricle width
MRI: Magnetic resonance imaging
MW-AH-LV: Maximum width of the anterior horn of the lateral ventricle
TD-ChP-LV: Transverse diameter of the choroid plexus of the lateral ventricle
MAED: Maximum external diameter
MAED-Cr: Maximum external diameter of cranium
MAID-Cr: Maximum internal diameter of cranium
SD: Standard deviation
IQR: Interquartile range
